# A synthetic indicator on the impact of COVID-19 on the community’s health

**DOI:** 10.1371/journal.pone.0238970

**Published:** 2020-09-11

**Authors:** Carmen Herrero, Antonio Villar

**Affiliations:** 1 Department of Economics, University of Alicante, Alicante, Spain; 2 Department of Economics, Universidad Pablo de Olavide, Seville, Spain; Azienda Ospedaliero Universitaria Careggi, ITALY

## Abstract

The expansion of Covid-19 has severely hit the community’s health all over the world, killing hundreds of thousands of people, subjecting health systems to an enormous stress (besides derailing economic activities and altering personal and social behavior). Two elements are essential to monitor the evolution of the pandemic as well as to analyze the effectiveness of the response measures: reliable data and useful indicators. We present an indicator that helps to assess the impact of Covid-19 on the community’s health, combining two different components: the extent of the pandemics (i.e. the share of the population affected) and its severity (the intensity of the disease on those affected). The severity measure derives from the application of an evaluation protocol that allows comparing population distributions based on the proportions of those affected with different health conditions. We illustrate the functioning of this indicator over a case study regarding the situation of the Italian regions on March 9 (the beginning of the confinement) and April 8, 2020, one month later.

## 1. Introduction

The speed and spread of transmission of COVID-19 have forced governments all over the world to implement strong defensive measures to control the expansion of the epidemics and avoid the collapse of their health care systems. Assessing the effectiveness of those measures calls for surveillance strategies on its application and continuous monitoring of the disease’s evolution. Both aspects require the availability of reliable data and adequate evaluation protocols that transform those data into helpful indicators [1]. Several variables measure particular instances of the pandemics (e.g. reproduction rate, mortality rate, positive rate). Here we shall focus on the overall evaluation of the impact of Convid-19 on the community’s health.

According to the general recommendations of the World Health Organization [2], there are three variables to consider in a pandemic of this nature: how many people are infected (transmissibility), how severely sick get the infected individuals, and how the pandemic affects the health-care system and society. In a similar vein, the US Department of Health and Human Services has developed the Pandemic Severity Assessment Framework (PSAF) [3]. The PSAF proposes two assessment dimensions, transmissibility, and clinical severity, and distinguishes on how to apply those measurement protocols depending on the stage of the epidemic [4]. The European Centre for Disease Prevention and Control (ECDC) has recently advised to “monitor the intensity, geographic spread and severity of COVID-19 in the population to estimate the burden of disease, assess the direction of recent time trends, and inform appropriate mitigation measures” [5]. The ECDC recommends countries to comprehensively testing suspected cases and to report the number of confirmed cases, distinguishing between those hospitalized, those in intensive care units (ICU), and those deceased.

There is, therefore, an extensive agreement on the variables that should be considered to track the evolution and the impact of COVID-19 on community’s health. There is also consensus on the way of approaching the evaluation, which can be regarded as a conventional way to assess the global impact of a given phenomenon on a population: computing both extent (the share of people involved) and severity (how intensely the event affects that population).

We know that countries have only been computing a fraction of the people infected by the virus, especially at the beginning of the pandemics, depending on detection policies and test availability [6]. Something similar can be said of the reports on the numbers of people dead and cured, as there is evidence that different countries (or even different regions within a country) apply diverse protocols to compute those cases. As a result, we do not have an exact picture of the dynamics of the disease [7]. Yet, it is still essential to get an idea on how things are evolving [8], if only to determine the effectiveness of the solutions that are being implemented, helping to calculate the needs of sanitary supplies, the pressure on the equipment and human resources of the health systems, and predicting the evolution of the disease and the progressive return to normality. Hence the need to tackle the second challenge: finding adequate indicators of the extent and severity of the pandemic.

Evaluating the severity of the disease requires analyzing the distribution of the population affected in different health conditions (e.g., hospitalized, in intensive care units, recovered, deceased). Ideally, we would like to have a numerical indicator that allows for *quantitative* comparisons to assess both the direction and the size of the changes in severity. This involves the design of an evaluation formula that, as a general rule, adopts the structure of a weighted average or a generalized mean of the relative frequencies of those health conditions. That is, we need to assign weights to each of those conditions and decide, for instance, how much we value the death of one person relative to the healing of another. Our conclusions on the severity of the pandemics will depend on those judgments, which are extremely difficult to determine for both technical and ethical reasons.

Herrero and Villar [9, 10] have developed an evaluation procedure that does not require introducing those judgments, which can help to assess the severity of the impact of COVID-19. Relying on the comparison of the probabilities that members of a community being worse-off than members of some other, we obtain a cardinal measure of the relative severity with which the pandemic affects different populations. Note that populations here may refer to the people infected in a group of countries, regions within a country, demographic groups, or different points in time. We can thus apply this evaluation procedure to estimate the impact on the community’s health of COVID-19 in a variety of ways.

We organize the paper as follows. Section 2 presents the evaluation protocol, which consists of the product of a measure of the extent and a measure of the severity. Section 3 illustrates this evaluation protocol regarding the situation of Italy and its regions in two points in time: the beginning of the confinement and one month later. Finally, Section 4 contains a short discussion on some of the critical aspects of this evaluation procedure and its applicability.

## 2. The proposal

### 2.1 The indicator

We propose to measure the impact of COVID-19 by an indicator made out of two components: *extent* (share of the population affected by the virus) and *severity* (a measure of the relative health situation of that population). The indicator, denoted by *I*_*Co*19_, consists of the product of those two variables. That is, *I*_*Co*19_ = *Extent*×*Severity*.

More formally, if we call $n_{h}^{a}$ the population affected by the virus in society *h*, *n*_*h*_ the total population of that society, and *s*_*h*_ the measure of severity, our indicator for population *h* is given by:

$$I_{Co19}(h)=\frac{n_{h}^{a}\times s_{h}}{n_{h}}$$

The indicator provides an intuitive measure of the degree to which each community is affected by the disease, as it describes how many people are affected, times how severely affected they are, relative to the population size. This is a standard measurement rod to estimate the impact of a given phenomenon on a population subgroup; in particular, it is the conventional approach regarding poverty measures [11, 12].

Note that we have used the expression “population affected by the virus” for $n_{h}^{a}$, rather than “population infected”, and “extent” for $n_{h}^{a}/n_{i}$, rather than “incidence”. The reason is that, especially during the initial phase of the contagion, those registered as infected were only those who required some kind of medical treatment or preventive measure (e.g. isolation). Monitoring is nowadays more thorough and present data also capture people with light or no symptoms. Needless to say, the indicator works with whatever reference population we consider, as its internal logic is independent of that aspect. Yet, the interpretation of the results is conditional on that reference population. We shall be precise on this respect in the illustration presented in Section 3 and will say more on this subject in the discussion (Section 4).

### 2.2 The evaluation protocol for severity

Let us now address the question of how to measure the severity. The formal problem consists of comparing a collection of populations affected by the virus, *G* = {1, 2, …, g}, in terms of the distributions of their members over an ordered set of health conditions, *c* = 1, 2, …, *C*. We describe the health situation of population *h* by a vector ***a***(*h*) = (*a*_*h*1_, *a*_*h*2_,…,*a*_*hC*_), where *a*_*hc*_ is the fraction of people affected by the virus in population *h* with health condition *c*. That is, we can write $a_{hc}=n_{h}^{ac}/n_{h}^{a}$, where $n_{h}^{ac}$ is the number of individuals in population *h* who are affected by the virus and exhibit health condition *c*. By construction, $a_{hc}\geq 0,\sum_{c=1}^{C}a_{hc}=1$, for all *h*.

To assess the relative situation of those populations, regarding the intensity of the pandemics, we compare the likelihood of getting a worse health condition for representative members of those societies. To be precise, let *p*_*hk*_ denote the probability that a member of society *h* exhibits a worse health condition than a member of society *k*. Assuming that those categories are ordered from worst to best, such a probability obtains as follows:

$$p_{hk}=a_{h1}(a_{k2}+\cdots +a_{kC})+a_{h2}(a_{k3}+\cdots +a_{kC})+\cdots +a_{h(C-1)}a_{kC}$$

Let *e*_*hk*_ = *e*_*kh*_ stand for the probability of a tie and define $q_{hk}=p_{hk}+\frac{1}{2}e_{hk}$ (i.e., we split the probability of a tie evenly).

To compare the severity of the pandemics in two societies, *h*, and *k*, we apply the following principle: the severity measures of those societies are proportional to the corresponding probabilities of being relatively worse off. That is if we call *s*_*h*_, *s*_*k*_ the severity measures, we let:^9,10^

$$\frac{s_{h}}{s_{k}}=\frac{q_{hk}}{q_{kh}}$$

Note that, by construction, this equation has a degree of freedom. That is, we can freely set the units in which we measure severity. In the two-society case, by letting *s*_*h*_ = 1, we can rewrite the former equation as follows:

$$s_{h}=\frac{q_{hk}}{{1-q}_{hk}}$$

That is, *s*_*h*_ tells us how likely is that an individual from society *h* is in a worse health condition than a individual from society *k*, relative to the complementary case.

When there are more than two societies involved this simple formulation has to be adjusted. Yet, we can extend this principle easily by taking expectations over the expression $s_{h}=\frac{q_{hk}}{q_{kh}}s_{k}$ (we cannot rely on bilateral comparisons as they are not transitive). That is, we measure severity in society *h* relative to the rest by the following formula:

$$s_{h}=\frac{\frac{1}{g-1}\sum_{k\neq h}q_{hk}s_{k}}{\frac{1}{g-1}\sum_{k\neq h}\left(1-q_{hk}\right)}$$

The previous expression has a similar meaning as before, even though now each probability in the numerator is weighted by the corresponding measure of severity.

The vector or those *s*_*h*_ values is called the *balanced worth* [10] and obtains as the dominant eigenvector of a Perron matrix **P** built as follows. The elements in the diagonal are of the form *R*_*k*_ = (*g*−1)−∑_*h*≠*k*_ *q*_*kh*_; the off-diagonal elements are just the probability values *q*_*hk*_. That is,

$$P=\left(\begin{array}{c} R_{1}\;q_{12}\;\ldots \;q_{1(g-1)}\;q_{1g}\\ q_{21}\;R_{2}\;\ldots \;q_{2(g-1)}\;q_{2g}\\ \ldots \;\ldots \;\ldots \;\ldots \\ q_{g1}\;q_{g2}\;\ldots \;q_{g(g-1)}\;R_{g} \end{array}\right)$$

The balanced worth provides a *relative evaluation* of the severity of the pandemic in the different populations considered. The structure of this matrix ensures that the balanced worth vector ***s*** = (*s*_1_,…,*s*_*g*_), which corresponds to the solution to the equation **s** = **Ps**, always exists, and it is positive and generically unique, except for the choice of units, as it has one degree of freedom. Thus, we have to normalize those values with respect some reference level that will define the units in which we measure this variable.

**Remark.** There is a friendly and freely accessible algorithm hosted in the Ivie website https://web2011.ivie.es/balanced-worth/balanced-worth-vector.php that performs instantly all calculations required to obtain this vector. This algorithm uses the mean, by default, to normalize the values of the corresponding eigenvector (i.e. we measure the values obtained in terms of percentages of the mean value). This normalization can be easily modified.

## 3. A case study: The impact of Covid-19 on the Italian regions

Let us see how this evaluation protocol works in a case study. This section serves the purpose of illustrating the application of the methodological approach we propose, rather than to provide an empirical study. We consider two different applications that show the measurement we can obtain in a synchronic and in a diachronic context. We first compare the situation of the Italian regions on April 8, 2020, which is one month after Italy decreed the confinement. Here we measure the impact of the Covid-19 in the Italian regions relative to the whole country. With this exercise we capture the diversity of the situations in the Italian regions at a particular point in time. Then we address the change experienced by those regions between March 9, the day in which the confinement started in Italy, and April 8. Here we compare the situation of the Italian regions relative to the initial state (Italy as a whole on March 9), so that we can have an estimate of the evolution of the pandemics within the regions and also relate how diverse was the situation at the beginning of the confinement and one month later.

The data come from the Italian Ministry of Health (Ministero della Salute), which are freely available at its webpage [13]. They referred to the people infected by the virus who had been identified due to the gravity of their symptoms. They included those treated in hospitals, those who have been isolated at home, cured, or have died. There was not, at that early stage, any estimate of those who might be infected but showed no apparent symptoms. We refer to the population registered as infected in that period as *infected with worrisome symptoms* (IWS, for short).

### 3.1 A day in the life of Italy with Covid-19

Table 1 describes the cumulative number of the IWS population on April 8. Individuals in this group presented one of the following five health conditions, ordered from worst to best: deceased, in intensive care units (ICU), hospitalized (non-ICU), isolated at home, and cured.

**Table 1:** IWS by health state (Italian regions, April 8, 2020).

	Deceased	Intensive care	Hospital	Isolated at home	Cured	Total
**Abruzzo**	179	62	331	1141	146	1859
**Basilicata**	14	17	48	205	13	297
**Bolzano**	183	65	268	948	371	1835
**Calabria**	60	15	170	570	44	859
**Campania**	221	97	608	2154	188	3268
**Emilia Romagna**	2234	361	3769	8980	2890	18234
**Friuli V.G.**	169	41	162	1212	634	2218
**Lazio**	244	196	1241	2011	574	4266
**Liguria**	654	153	1109	1983	1007	4906
**Lombardia**	9722	1257	11719	15569	15147	53414
**Marche**	652	133	974	2455	645	4859
**Molise**	13	4	30	147	32	226
**Piemonte**	1378	423	3493	7073	1516	13883
**Puglia**	219	90	639	1509	177	2634
**Sardegna**	59	31	112	697	76	975
**Sicilia**	133	65	563	1265	133	2159
**Toscana**	392	260	1066	4231	430	6379
**Trento**	255	77	354	1509	407	2602
**Umbria**	50	41	155	627	416	1289
**Valle d'Aosta**	102	20	120	466	142	850
**Veneto**	736	285	1554	8332	1503	12410
**Total**	17669	3693	28485	63084	26491	139422

Source: Ministero della Salute (Italy)

Table 2 shows that Italian regions exhibited a large variability regarding the extent of the COVID-19 (see the last column of the table), with a coefficient of variation around 0∙8. The highest values were in the Northern regions: Lombardia, Emilia-Romagna, Piemonte, Marche, Liguria, Trento, Bolzano, and Val d’Aosta. We can decompose the extent figure into two components: the product of the ratio of the IWS over the number of tests performed, and the ratio between the number of tests per 100,000 inhabitants. The first term tells how the IWS relates to the number of tests (a measure of the detection rate). The second term is an index of how intense the search of IWS was between regions.

**Table 2:** IWS, tests, and population (Italian regions, April 8, 2020).

	IWS/Test	Test per 100,000 inhabs.	IWS per 100,000 inhabs.
**Abruzzo**	11∙73%	1208	142
**Basilicata**	9∙01%	586	53
**Bolzano**	9∙73%	3552	345
**Calabria**	5∙74%	769	44
**Campania**	11∙76%	479	56
**Emilia Romagna**	23∙27%	1757	409
**Friuli V.G.**	8∙94%	2041	183
**Lazio**	7∙74%	937	73
**Liguria**	28∙00%	1130	316
**Lombardia**	31∙88%	1665	531
**Marche**	27∙72%	1149	319
**Molise**	11∙29%	655	74
**Piemonte**	28∙63%	1113	319
**Puglia**	10∙75%	608	65
**Sardegna**	11∙48%	518	59
**Sicilia**	7∙87%	549	43
**Toscana**	10∙46%	1635	171
**Trento**	19∙63%	2450	481
**Umbria**	9∙14%	1599	146
**Valle d'Aosta**	28∙78%	2350	676
**Veneto**	7∙60%	3328	253
**Total**	17∙27%	1337	231

Source: Ministero della Salute (Italy)

Data show that the more intense the search, the more cases detected (a coefficient of correlation of 0∙624). Despite the variability of the ratio between IWS and the number of tests performed, the extent variable is orthogonal with that measuring the tests per 100.000 inhabitants (a correlation coefficient of 0∙1). That is, data suggest that the differential impact of the disease over the regions was not due to the differential search intensities.

**Fig 1:**
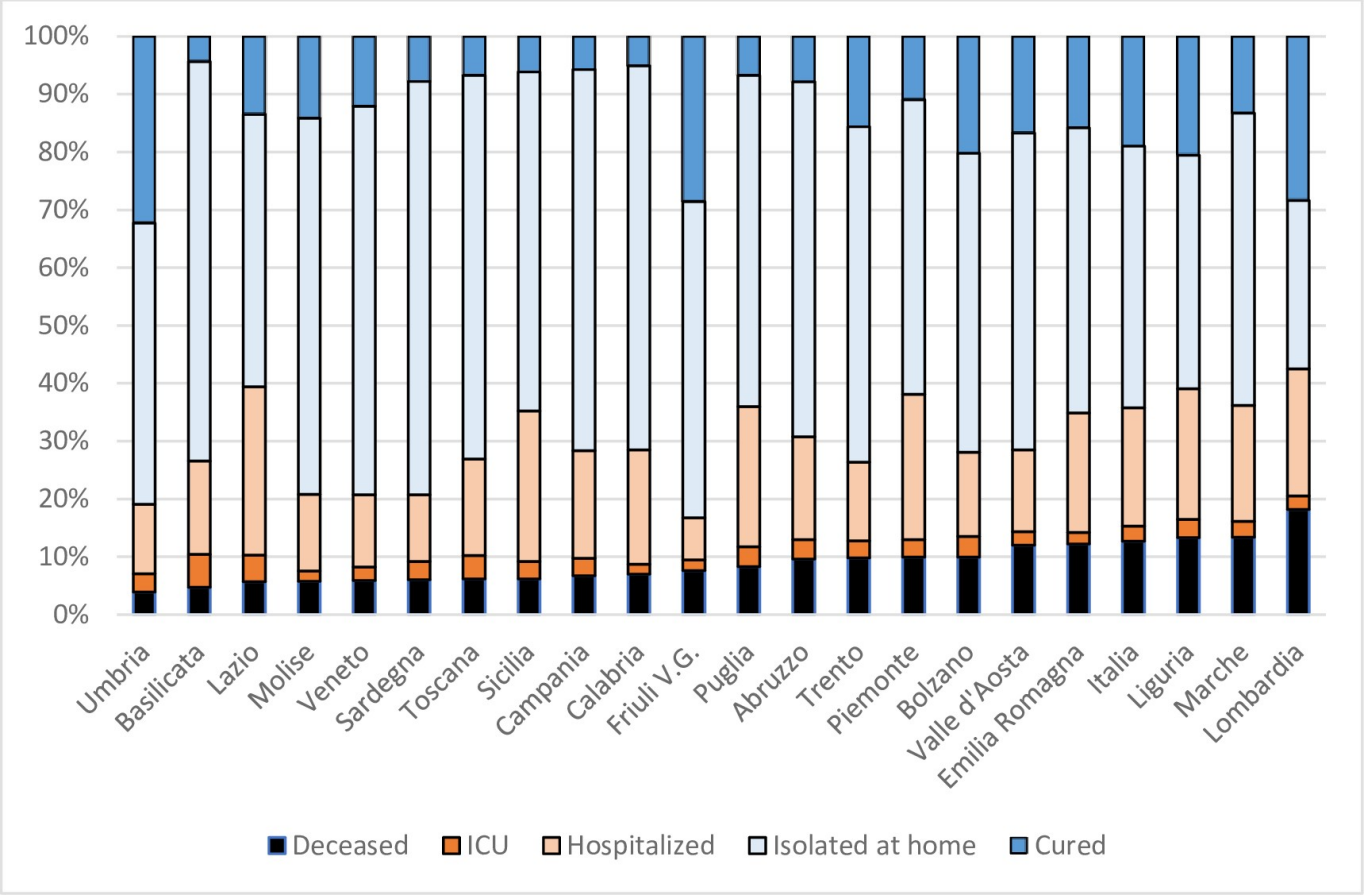
IWS shares by health condition (Italian regions, April 8, 2020). Source: Ministero della Salute (Italy).

Fig 1 displays the proportions of the IWS population into the different health conditions (arranged by increasing number of deceased). The proportions of those deceased and cured presented a large variability (with coefficients of variation of 0·4 and 0·57, respectively). For those isolated at home, the variability was relatively low (CV = 0·18), whereas that of those hospitalized or at the ICU was somewhere in between (CV = 0·3 in both cases).

Fig 1 illustrates well the challenge of transforming those data into an indicator of severity and gives a hint on how things can appear depending on the way of attaching values to the health conditions. To obtain the severity measure described in Section 2 (the so-called *balance worth*), we just have to plug the data generating this figure into the web page mentioned in the Remark above. This measure tells us about the relative health situation of the IWS in the Italian regions. To facilitate the comparison, we normalize the values by setting Italy to 100. Table 3 reports the evaluation of the severity of COVID-19. There are two features worth commenting. First, the variability was relatively small, with a coefficient of variation of 0∙155. Second, some of the regions with higher severity were in the South, where the extent was much smaller.

**Table 3:** Severity and impact of COVID-19 in the Italian regions (April 8, 2020).

	Severity	Impact COVID-19
**Abruzzo**	106∙63	65∙43
**Basilicata**	102∙81	23∙49
**Bolzano**	86∙45	129∙30
**Calabria**	104∙52	19∙96
**Campania**	103∙71	25∙29
**Emilia Romagna**	102∙60	181∙62
**Friuli V.G.**	63∙01	49∙79
**Lazio**	109∙86	34∙51
**Liguria**	103∙50	141∙77
**Lombardia**	100∙56	231∙14
**Marche**	109∙68	151∙27
**Molise**	81∙33	26∙04
**Piemonte**	114∙15	157∙49
**Puglia**	116∙20	32∙89
**Sardegna**	89∙57	23∙06
**Sicilia**	113∙93	21∙30
**Toscana**	100∙21	74∙20
**Trento**	89∙47	186∙26
**Umbria**	60∙64	38∙37
**Valle d'Aosta**	92∙08	269∙64
**Veneto**	83∙95	91∙94
**Total**	100	100

Source: Ministero della Salute (Italy) and own calculations.

The indicator we propose to measure the impact of COVID-19 over the community’s health simply obtained by weighing extent by severity. The resulting data appear in Table 3. The variability of the impact was extremely high, with a coefficient of variation above 0∙8. Lombardia and Valle d’Aosta presented the highest impact, followed by Trento and Emilia-Romagna. The lowest impact corresponded to Sicilia, Calabria, and Basilicata.

### 3.2 Changes after one month of confinement

We now discuss how the situation changed between March 9 and April 8. Table 4 provides the relevant information for those two dates, setting Italy to 100 on March 9, both for severity and impact. There are several features worth commenting.

**Table 4:** Severity and impact in the Italian regions on March 9 and April 8 (Italia = 100 on March 9 for both variables).

	Severity March 9	Severity April 8	Impact March 9	Impact April 8
**Abruzzo**	138	58	21	541
**Basilicata**	68	56	4	194
**Bolzano**	149	51	17	1159
**Calabria**	97	58	4	168
**Campania**	74	58	10	215
**Emilia-R.**	93∙5	63∙5	191	1709
**Friuli V.G.**	51	36	26	432
**Lazio**	116	66	13	315
**Liguria**	146∙5	70∙5	68	1468
**Lombardia**	105∙4	73∙3	377	2561
**Marche**	111	69	155	1447
**Molise**	82	46	25	224
**Piemonte**	159∙6	68∙6	84	1439
**Puglia**	107∙4	68	9	293
**Sardegna**	71	48	5	188
**Sicilia**	60	64	4	182
**Toscana**	92∙6	55∙1	34	620
**Trento**	69	52∙3	28	1655
**Umbria**	55∙3	34∙6	12	333
**V. d'Aosta**	56∙2	53∙8	44	2395
**Veneto**	66	47∙2	66	786
**Total**	100	64∙7	100	983

Source: Ministero della Salute (Italy) and own calculations.

During this month the impact in the whole country multiplied by a factor of 10, whereas in some regions multiplied by more than 40 times: Bolzano (70 times), Trento (60 times), Valle d’Aosta (54 times), Basilicata (49 times), Calabria (47 times), and Sicilia (43 times). All these regions exhibited low impact values on March 9 (especially the last three regions). The regions with a higher impact on March 9 display much smaller factors: Lombardia (6 times), Marche (8 times), Veneto (12 times), Piamonte (17 times), Liguria (18 times). As a consequence, the extreme diversity between the Italian regions, as measured by the coefficient of variation, exhibits a substantial reduction between March and April (from 0∙347 to 0∙185 for severity and from 1∙519 to 0∙853 for impact). This fact may indicate that confinement is an effective policy in fighting the disease.

Severity decreased substantially in most of the regions, with an overall reduction of 33%. This reduction happened more intensely in those regions with worse indicators so that we observe a sharp decline in its variability, which dropped by almost one half. This suggests that the health system was responding correctly, and did it more intensely in those regions more in need.

## 4. Discussion

Many countries provide daily reports on the effects of COVID-19 regarding the spread of the infection, the numbers of people dead, hospitalized, in intensive care units, and cured. Those data evolve differently both within each population (they increase and decrease and do it at different rates) and between populations (e.g., countries, regions, age groups). This complex dynamics makes it challenging to get an idea of the global impact of COVID-19 on community’s health. We have presented a protocol intended to address this evaluation problem. It measures impact as the product of extent and severity. Extent is simply the share of those registered as infected in the population whereas severity is a more sophisticated indicator.

### 4.1 Severity

Severity is measured by comparing distributions across different health conditions of the populations affected by the virus. The type of comparison proposed here (the balanced worth) permits one to get a cardinal measure without having to attach weights to those health conditions. We depart, therefore, from other approaches based on setting ex-ante scores to those states (e.g. the weights used to ponder different health states in an advanced phase of the epidemics in PSAF) and on the “disability-adjusted life years” metrics used to estimate the “burden of disease.” The nature of the available information at that early stage of contagion makes it difficult to apply those evaluation formulae [4].

This severity measure is based on the relative likelihood of getting a worse health condition for a representative member of an affected population. The formula is intuitive and corresponds to a well-known mathematical tool, similar to the one used by Google to order web pages or the principle behind the Eigenfactor [14, 15]. The evaluation obtains as the dominant eigenvector of a Perron matrix associated with a Markov chain [16]. Therefore, calculations are conventional, and we know precisely how the evaluation protocol works and what information conveys. With the advantage of providing quantitative estimates and having designed a specific algorithm that is freely available.

**Fig 2:**
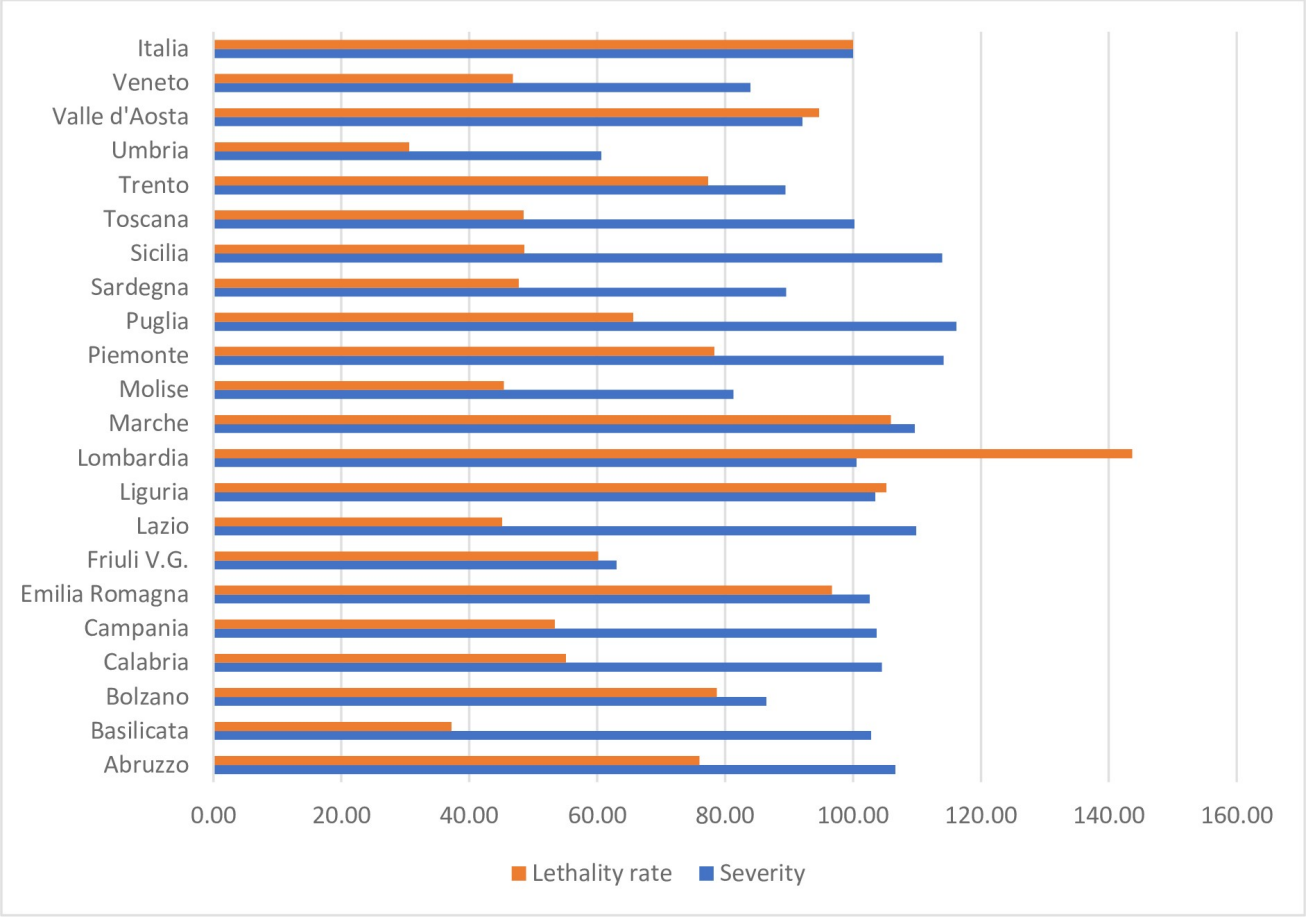
Lethality rate and severity in the Italian regions, on March 9 (Italy = 100 for both variables). Source: Ministero della Salute (Italy).

It is worth mentioning that severity is not a variant of another elementary indicator, such as the lethality rate (i.e., the ratio between deceased and affected). Fig 2 illustrates this fact by comparing severity and lethality in the Italian regions on April 8, normalizing both rates setting Italy to 100. The correlation coefficient is below 0·28.

### 4.2 Population subgroups

Our way of comparing distributions implies that the evaluation is relative. That is, we obtain an assessment of how a population fares relative to others. This fact is essential both to understand the meaning of the evaluation and to think of the different questions this protocol permits to address. Besides the types of the evaluation presented here, regarding the comparison of different populations (Italian regions) at a given point in time and different dates, we may consider different types of individuals (depending on age, gender, race, wealth, etc.) or particular population subgroups [10].

A population subgroup of particular relevance is that corresponding to those who are positive at the reference day, that is, those registered as infected who are in intensive care units, at the hospital or isolated at home. Let us call PAP this population subgroup, as a shorthand for Positive At Present. The impact of Covid-19 over the PAP population is a measure of the effort currently required from the health system, as we discount from the population affected those already cured and those deceased.

**Fig 3:**
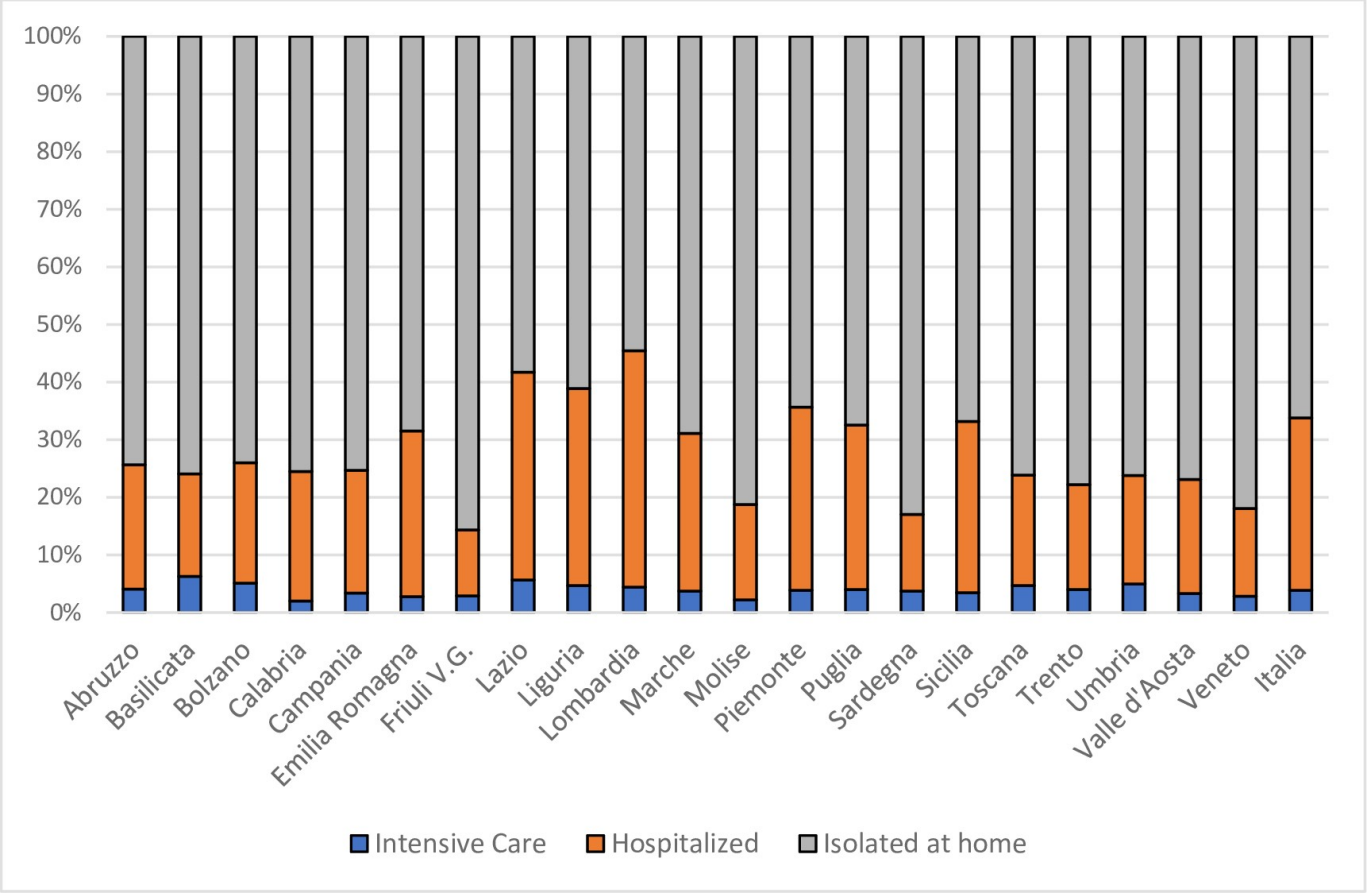
PAP shares by health condition (Italian regions, April 8, 2020). Source: Ministero della Salute (Italy).

Fig 3 describes the shares of the PAP on April 8 in the Italian regions, using the data in Table 1. They show that Lazio, Liguria, Lombardia and Piemonte were the regions with higher shares of people in hospitals (including those in ICU). Those with smaller shares corresponded to Friuli V.G., Molise, Sardegna and Veneto.

Table 5 provides the evaluation of the Italian regions on April 8, in terms of severity and impact, for the PAP population. As it was the case for the IWS population, the impact has much larger variability than the severity (a coefficient of variation of 0.759 with respect to 0.166). Valle d’Aosta, Lombardia, Trento, Emilia-Romagna, Liguria and Bolzano were the regions with a more substantial impact of Covid-19 on the PAP. Calabria, Campania, Molise and Sicilia were those with a smaller impact.

**Table 5:** Severity and impact of COVID-19 in the population of positive on April 8, 2020. Italian regions.

	Severity	Impact
**Abruzzo**	85∙48	63∙34
**Basilicata**	84∙04	25∙54
**Bolzano**	86∙61	132∙35
**Calabria**	82∙68	20∙31
**Campania**	83∙59	26∙10
**Emilia Romagna**	95∙08	177∙11
**Friuli V.G.**	68∙17	50∙30
**Lazio**	117∙88	43∙81
**Liguria**	110∙99	147∙17
**Lombardia**	126∙10	226∙70
**Marche**	94∙83	140∙32
**Molise**	74∙07	27∙80
**Piemonte**	103∙66	165∙68
**Puglia**	97∙77	34∙41
**Sardegna**	72∙18	23∙43
**Sicilia**	98∙59	23∙65
**Toscana**	82∙92	78∙28
**Trento**	79∙99	181∙72
**Umbria**	82∙99	49∙07
**Valle d'Aosta**	81∙06	247∙69
**Veneto**	73∙31	96∙30
**Italia**	100	100

Source: Ministero della Salute (Italy) and own calculations.

It is also interesting to observe how severity has changed along this month by comparing our two reference dates and anchoring the evaluation by setting Italy to 100 on March 9, as shown in Table 6. There are two remarkable features that those data reveal. First∙ the sharp decline of the severity values in all regions, to almost one half of the initial value for the whole country. Second, the even sharper reduction of the variability between regions (60% reduction in the coefficient of variation. which drops from 0∙462 to 0∙168). That suggests, once more, that the health system reacted in a balanced way absorbing the shock according to need.

**Table 6:** Severity in the PAP population on March 9 and April 8 2020. Italian regions.

	Mar-09	Apr-08
**Abruzzo**	140.42	44∙47
**Basilicata**	57∙26	37∙70
**Bolzano**	159∙81	44∙09
**Calabria**	159∙81	41∙85
**Campania**	63∙72	62∙64
**Emilia Romagna**	77∙63	49∙55
**Friuli V∙G∙**	39∙38	43∙29
**Lazio**	107∙48	58∙68
**Liguria**	154∙91	41∙37
**Lombardia**	121∙58	66∙71
**Marche**	96∙05	43∙15
**Molise**	69∙76	43∙25
**Piemonte**	155∙17	37∙37
**Puglia**	90∙56	34∙45
**Sardegna**	59∙73	36∙88
**Sicilia**	53∙39	51∙30
**Toscana**	82∙85	49∙61
**Trento**	56∙53	51∙62
**Umbria**	42∙08	45∙30
**Valle d'Aosta**	43∙56	42∙51
**Veneto**	54∙77	54∙49
**Total**	100	52∙50

Source: Ministero della Salute (Italy) and own calculations.

### 4.3 The dynamics of the pandemics

There is a strong suspicion that, at the initial phase of the pandemics, the number of people infected was much larger than reported. The multiplication of the tests and the surveys on seropositivity have started capturing people infected with light or no symptoms so that nowadays data are richer and more informative.^13^ Those data might induce a revision of the extent and severity of the pandemics in that initial phase, recurring to some statistical techniques. Indeed, there is already some statistical analysis on the “excess of deaths”, which suggests that the number of people registered as deceased by the virus was also underestimated [17]. As a consequence, some revisions on the data on the evolution of the pandemics are to be expected.

The new data available entail a change in the nature of the reference population and has implications on the impact analysis. Measuring severity in this richer scenario will require introducing another health condition, corresponding to infected people with light or no symptoms. As severity is a relative measure, the effect of introducing this new category will depend on the distribution of those infected but asymptomatic between the populations under consideration.

Introducing that new health condition is a trivial change in the analysis presented in Section 3. Note that, in a synchronic analysis, this modification presents no particular problem. Things are different in a diachronic analysis because the change in the population registered as infected in March 2020 and in September 2020, say, involves an implicit change in the criterion that defines those who are infected. It would thus be prudent, for the time being, to analyze the evolution of the impact within periods in which the recording criteria have not changed much, or keeping as the reference population those infected with worrisome symptoms (the IWS population used in our empirical application), Alternatively, one may smooth the effect of the change in the detection policy by making the impact analysis on a very short period bases (e.g. daily) using a moving average approach [18, 19].

## Supporting information

S1 File (DOCX)
