# Supplementary material for: A synthetic indicator on the impact of COVID-19 on the community’s health
Source: PLoS One. 2020 Sep 11;15(9):e0238970. doi: 10.1371/journal.pone.0238970 (PMC7485889; doi:10.1371/journal.pone.0238970)
Supplement: S1 File — (DOCX) [file pone.0238970.s001.docx]

WORKABLE FILES WITH ORIGINAL DATA (All of them publicly available at the Italian Ministry of Health in pdf format).

| **AGGIORNAMENTO 09/03/2020 ORE 17.00** | | | | | |  |  |  |
| --- | --- | --- | --- | --- | --- | --- | --- | --- |
| Regione | POSITIVI AL nCoV | |  |  |  |  |  |  |
|  | Ricoverati con sintomi | Terapia intensiva | Isolamento domiciliare | Totale attualmente positivi | DIMESSI GUARITI | DECEDUTI | CASI TOTALI | TAMPONI |
| Lombardia | 2802 | 440 | 1248 | 4490 | 646 | 333 | 5469 | 20135 |
| Emilia Romagna | 576 | 90 | 620 | 1286 | 30 | 70 | 1386 | 4906 |
| Veneto | 186 | 51 | 457 | 694 | 30 | 20 | 744 | 15956 |
| Piemonte | 222 | 50 | 65 | 337 |  | 13 | 350 | 1681 |
| Marche | 136 | 47 | 130 | 313 |  | 10 | 323 | 1250 |
| Toscana | 107 | 9 | 90 | 206 | 1 | 1 | 208 | 2018 |
| Lazio | 55 | 8 | 31 | 94 | 3 | 5 | 102 | 1929 |
| Campania | 42 | 8 | 69 | 119 | 1 |  | 120 | 980 |
| Liguria | 60 | 17 | 20 | 97 | 5 | 7 | 109 | 611 |
| Friuli V.G. | 18 | 1 | 70 | 89 | 3 | 1 | 93 | 1344 |
| Sicilia | 19 |  | 33 | 52 | 2 |  | 54 | 836 |
| Puglia | 20 | 6 | 20 | 46 | 1 | 3 | 50 | 685 |
| Trento | 10 | 2 | 21 | 33 |  |  | 33 | 267 |
| Abruzzo | 25 |  | 5 | 30 |  |  | 30 | 237 |
| Umbria | 4 | 2 | 22 | 28 |  |  | 28 | 183 |
| Molise | 4 | 2 | 8 | 14 |  |  | 14 | 212 |
| Sardegna | 8 |  | 11 | 19 |  |  | 19 | 185 |
| Valle d'Aosta | 4 |  | 11 | 15 |  |  | 15 | 67 |
| Calabria | 8 |  | 1 | 9 | 2 |  | 11 | 173 |
| Bolzano | 8 |  | 1 | 9 |  |  | 9 | 36 |
| Basilicata | 2 |  | 3 | 5 |  |  | 5 | 135 |
| TOTALE | 4316 | 733 | 2936 | 7985 | 724 | 463 | 9172 | 53826 |
| ATTUALMENTE POSITIVI | 7985 |  |  |  |  |  |  |  |
| TOTALE GUARITI | 724 |  |  |  |  |  |  |  |
| TOTALE DECEDUTI | 463 |  |  |  |  |  |  |  |
| CASI TOTALI | 9172 |  |  |  |  |  |  |  |

|  | **AGGIORNAMENTO 08/04/2020 ORE 17.00** | | | | | | | |
| --- | --- | --- | --- | --- | --- | --- | --- | --- |
| Regione | POSITIVI AL nCoV | | | | DIMESSI/ GUARITI | DECEDUTI | CASI TOTALI | TAMPONI |
|  | Ricoverati con sintomi | Terapia intensiva | Isolamento domiciliare | Totale attualmente positivi |  |  |  |  |
| Lombardia | 11719 | 1257 | 15569 | 28545 | 15147 | 9722 | 53414 | 167557 |
| Emilia Romagna | 3769 | 361 | 8980 | 13110 | 2890 | 2234 | 18234 | 78367 |
| Piemonte | 3493 | 423 | 7073 | 10989 | 1516 | 1378 | 13883 | 48495 |
| Veneto | 1554 | 285 | 8332 | 10171 | 1503 | 736 | 12410 | 163247 |
| Toscana | 1066 | 260 | 4231 | 5557 | 430 | 392 | 6379 | 60985 |
| Marche | 974 | 133 | 2455 | 3562 | 645 | 652 | 4859 | 17532 |
| Liguria | 1109 | 153 | 1983 | 3245 | 1007 | 654 | 4906 | 17521 |
| Lazio | 1241 | 196 | 2011 | 3448 | 574 | 244 | 4266 | 55113 |
| Trento | 354 | 77 | 1509 | 1940 | 407 | 255 | 2602 | 13258 |
| Campania | 608 | 97 | 2154 | 2859 | 188 | 221 | 3268 | 27784 |
| Puglia | 639 | 90 | 1509 | 2238 | 177 | 219 | 2634 | 24493 |
| Friuli V.G. | 162 | 41 | 1212 | 1415 | 634 | 169 | 2218 | 24798 |
| Sicilia | 563 | 65 | 1265 | 1893 | 133 | 133 | 2159 | 27438 |
| Abruzzo | 331 | 62 | 1141 | 1534 | 146 | 179 | 1859 | 15846 |
| Bolzano | 268 | 65 | 948 | 1281 | 371 | 183 | 1835 | 18865 |
| Umbria | 155 | 41 | 627 | 823 | 416 | 50 | 1289 | 14105 |
| Sardegna | 112 | 31 | 697 | 840 | 76 | 59 | 975 | 8493 |
| Calabria | 170 | 15 | 570 | 755 | 44 | 60 | 859 | 14977 |
| Valle d'Aosta | 120 | 20 | 466 | 606 | 142 | 102 | 850 | 2953 |
| Basilicata | 48 | 17 | 205 | 270 | 13 | 14 | 297 | 3296 |
| Molise | 30 | 4 | 147 | 181 | 32 | 13 | 226 | 2002 |
| TOTALE | 28485 | 3693 | 63084 | 95262 | 26491 | 17669 | 139422 | 807125 |
